# Multi-voxel pattern analysis for developmental cognitive neuroscientists

**DOI:** 10.1016/j.dcn.2025.101555

**Published:** 2025-03-25

**Authors:** João F. Guassi Moreira, Jennifer A. Silvers

**Affiliations:** a Department of Psychology, University of Wisconsin, Madison, USA; b Department of Psychology, University of California, Los Angeles, USA

**Keywords:** Multi-voxel pattern analysis, FMRI, Decoding, Representational similarity analysis, Pattern expression, Voxel-wise encoding

## Abstract

The current prevailing approaches to analyzing task fMRI data in developmental cognitive neuroscience are brain connectivity and mass univariate task-based analyses, used either in isolation or as part of a broader analytic framework (e.g., BWAS). While these are powerful tools, it is somewhat surprising that multi-voxel pattern analysis (MVPA) is not more common in developmental cognitive neuroscience given its enhanced ability to both probe neural population codes and greater sensitivity relative to the mass univariate approach. Omitting MVPA methods might represent a missed opportunity to leverage a suite of tools that are uniquely poised to reveal mechanisms underlying brain development. The goal of this review is to spur awareness and adoption of MVPA in developmental cognitive neuroscience by providing a practical introduction to foundational MVPA concepts. We begin by defining MVPA and explain why examining multi-voxel patterns of brain activity can aid in understanding the developing human brain. We then survey four different types of MVPA: Decoding, representational similarity analysis (RSA), pattern expression, and voxel-wise encoding models. Each variant of MVPA is presented with a conceptual overview of the method followed by practical considerations and subvariants thereof. We go on to highlight the types of developmental questions that can be answered by MPVA, discuss practical matters in MVPA implementation germane to developmental cognitive neuroscientists, and make recommendations for integrating MVPA with the existing analytic ecosystem in the field.

## 1 Introduction

Developmental cognitive neuroscience has grown rapidly in the 21st century, in large part due to the advent and widespread adoption of functional magnetic resonance imaging (fMRI). The proliferation of fMRI has prompted increasing analytic sophistication in developmental cognitive neuroscience. Currently, the dominant analytic foci in the field for fMRI-based studies are functional connectivity and univariate task-based analyses, used either in isolation or as part of a broader analytic framework (e.g., brain wide association studies (BWAS)). By contrast, far less developmental cognitive neuroscience research utilizes multi-voxel pattern analysis (MVPA). This is somewhat surprising, given that MVPA has a unique potential to shed insight on core developmental processes that are more difficult to observe using univariate or connectivity methods. The goal of this article is to raise awareness of the developmental utility of MVPA, review four core variants of MVPA (decoding, representational similarity analysis, pattern expression, and voxel-wise encoding models), provide practical details for developmental cognitive neuroscientists using these methods, and propose points of extension and integration between MVPA and popular analytic techniques in the field.

### 1.1 Multi-voxel pattern analysis can aid developmental research but is underutilized in the field

As in related fields, research in developmental cognitive neuroscience generally unfolds in one of two broad categories. *Basic science* research aims to uncover foundational mechanisms that undergird brain development, for example such as how changes in brain structure affect brain function and behavior (Levakov et al., 2021), or how changing neural circuitry drives socioaffective phenomena in childhood and adolescence (Gee et al., 2013; Somerville et al., 2013). Basic developmental cognitive neuroscience research may also help evaluate theories from other adjacent fields (e.g., psychology, cognitive science), such testing dual systems models of decision-making (Cosme et al., 2019). Research in the *translational-applied* category is geared towards identifying biomarkers (Marek and Laumann 2024; Rapuano et al., 2020), building predictive models of clinically-relevant outcomes (e.g., substance use disorder, depression), identifying translationally actionable mechanisms (e.g., neural circuits amenable to clinical intervention; Aaden et al., 2021; Webler et al., 2024), charting population-based trends for informing policy (Falk et al., 2013; Wolf and Felsen 2019; Gell et al., 2025), or using insights from brain development for commercial-industrial purposes (Wager et al., 2013; Koban et al., 2023). What both basic and translational research the field have in common is that they care about developmental mechanisms and prediction. Knowledge of developmental mechanisms in developmental cognitive neuroscience is predicated on understanding how the brain changes in response to environmental demands, internal imperatives (e.g., genetic programming), and the interplay between the two (Smith and Thelen, 2003). By extension it is insufficient to simply track change over time (Poldrack, 2015)–it is necessary to understand why change is occurring or what is driving it. Prediction in the context of developmental neuroscience is based on being able to foretell some feature of a future developmental stage from a current, or past, stage in a sensitive and specific way (e.g., being able to confidently identify youth at risk of experiencing a maladaptive developmental outcome; establishing normative growth charts for neural phenotypes; e.g., Shafiei et al., 2025).

In this review, we argue that increased adoption of MVPA can be a boon to achieving these broad goals of developmental cognitive neuroscience. That is because MVPA is relatively well-suited for (i) revealing information, either directly or indirectly, about neural population coding, and (ii) tends to be more sensitive to task effects. The concept of neural population codes refers to the idea that behavior, cognition, and affect rely on the distributed and coordinated activity of many neuronal populations, each tuned to different stimuli and features thereof (Erickson,2001;Langdonetal.,2023;Panzerietal.,2015). MVPA allows researchers to learn more about population codes by probing how the brain represents information between relevant stimulus categories or mental states (to the extent possible with fMRI, see Freeman et al., 2011). This in turn offers developmental cognitive neuroscientists a relatively fine-grained understanding of how information and states are encoded in the brain, and examining how such codes change with age and experience may allow one to make more direct inferences about the catalyzing forces that drive development. In terms of prediction, MVPA tends to provide richer characterizations of data that are more sensitive to task effects, allowing researchers to engineer relatively more predictive models and thus more robust detection of individual difference associations between brain and behavior (Chang et al., 2015; Baranger et al., 2025, Davis et al., 2014, Hebart and Baker, 2018).

Despite this utility, MVPA is relatively under-utilized by researchers in developmental cognitive neuroscience. A coarse survey of conference programs from all twelve annual meetings of the Flux Congress (the academic society that publishes the field’s flagship journal, *Developmental Cognitive Neuroscience;* https://fluxsociety.org/) reveals that common MVPA related terms (‘MVPA’, ‘decod*’, ‘RSA’, and ‘representational’) were printed a combined 158 times from 2013 through 2024. By contrast, the terms ‘functional connectivity’ and ‘task’ appeared a combined 2066 times in that same span. Even though this survey did not account for negations and alternative uses of each term, the magnitude of this discrepany still underscores the relative underutilization of MVPA methods in the field. The underutilization of MVPA methods is curiously inconsistent with trends in the broader neuroscientific literature, which shows increases in the popularity of MVPA. Using the freely available Pubmed By Year tool (https://esperr.github.io/pubmed-by-year), we found that citation counts with the terms ‘representational similarity analysis fmri’ and ‘decoding fmri’ are positively correlated with publication year, to the degree of .93 and .97, respectively. This lends further evidence to the notion that MVPA is underutilized in developmental cognitive neuroscience.

### 1.2 Promoting MVPA adoption in developmental cognitive neuroscience

We suspect that one overarching reason driving the relative underutilization of MVPA in developmental cognitive neuroscience is a lack of awareness and accessibility. MVPA can be quite difficult to learn and thus inaccessible if one is not already part of a lab using MVPA techniques, or has not obtained formal instruction from a course or workshop. This issue is compounded by the fact that many in the field wish to link aspects of brain activity to age, developmental stage, or some developmentally-salient behavioral phenotype—MVPA may then feel even more inaccessible or complicated when one wants to incorporate it with additional information (to say nothing of integration with more advanced techniques such as longitudinal modeling or BWAS). Although several reviews of various individual MVPA methods already exist (Freund et al., 2021, Etzel et al., 2013, Weaverdyck et al., 2020, Popal et al., 2019, Naselaris et al., 2011, Mahmoudi et al., 2012; Cohen et al., 2017), there has yet to be one that is tailored specifically for developmental cognitive neuroscientists while also reviewing more than two variants of MVPA. With this review, we hope to provide interested developmental cognitive neuroscientists with an accessible introduction to foundational MVPA concepts. We accomplish this by (i) surveying four core variants of MVPA, (ii) outlining the types of developmental questions that can be answered with these variants of MVPA, (iii) identifying practical issues and providing tips for implementing MVPA in a developmental neuroscience context, (iv) and proposing novel ways to integrate MVPA with the field’s extant analytic ecosystem.

## 2 A survey of core multi-voxel pattern analysis techniques

Here we define MVPA and survey four core techniques that fall under its umbrella: decoding, representational similarity analysis (RSA), pattern expression analysis, and voxel-wise encoding models. While most other reviews of MVPA typically only cover decoding, RSA, or both, we seek to advance a ‘broadband’ definition of MVPA aligned with the theme of understanding neural population coding to the extent possible. Admittedly, MPVA methods need not strictly be used in search of identifying population codes, but the implicit, idealized goal of recovering a multi-dimensional encoding space encompasses the four aforementioned variants and is a useful categorizing heuristic. The survey includes a fifth sub-section on emerging techniques that fall under the MVPA umbrella but are not as widely used as the ‘core four’, and then concludes with a discussion of caveats and limitations.

### 2.1 Defining multi-voxel pattern analysis

Before defining MVPA, we need to define what is meant by “multi-voxel pattern” in this context. Colloquially, a pattern of neural activity simply could refer to any specific configuration of observed data or findings derived from brain activity. However, in in the context of MVPA, a multi-voxel pattern refers to fine-grained activity estimates (e.g., BOLD signal intensity, t-stats) from a meaningfully selected set of voxels. The key phrase ‘fine-grained’ necessitates an absence of much, or any, preprocessing that introduces additional artificial similarity between neighboring voxels (e.g., smoothing, spatial averaging). Following our definition of ‘multi-voxel pattern’, MVPA refers to any analysis that uses multi-voxel response patterns in some manner to understand how information is encoded in the brain. Leveraging the high dimensionality of voxel response patterns rather than averaging over them allows for better differentiation of stimuli or task-states (Kriegeskorte and Kievit, 2013; Mahmoudi et al., 2012), leaving MVPA relatively well-suited to answer questions about how these response patterns collectively comprise, or indirectly indicate, a code for some kind of information (e.g., stimulus categories, task states, behavioral performance) or for building more precise predictive models.

**Fig. 1:**
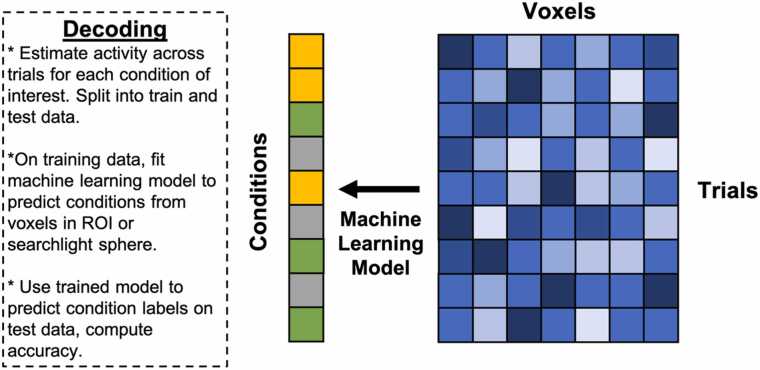
Decoding schematic.

**Fig. 2:**
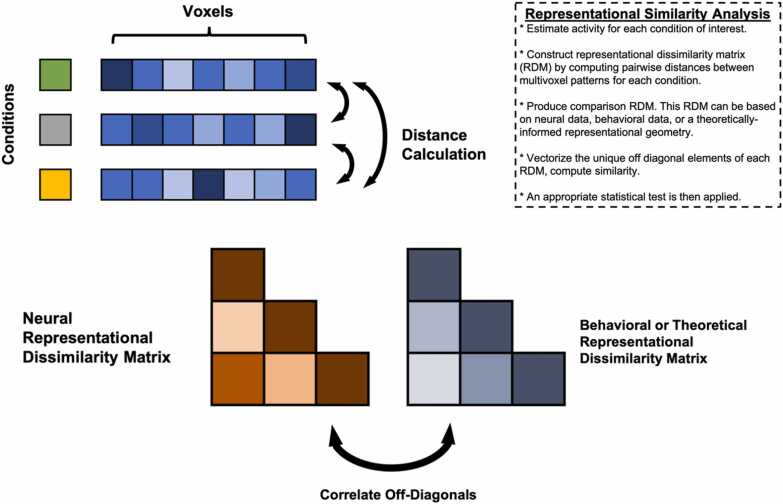
Representational Similarity Analysis Schematic.

**Fig. 3:**
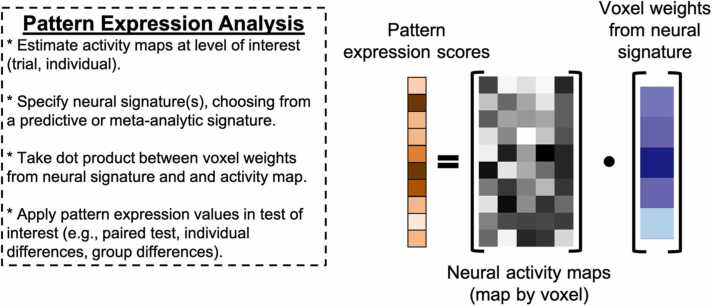
Pattern Expression Schematic.

**Fig. 4:**
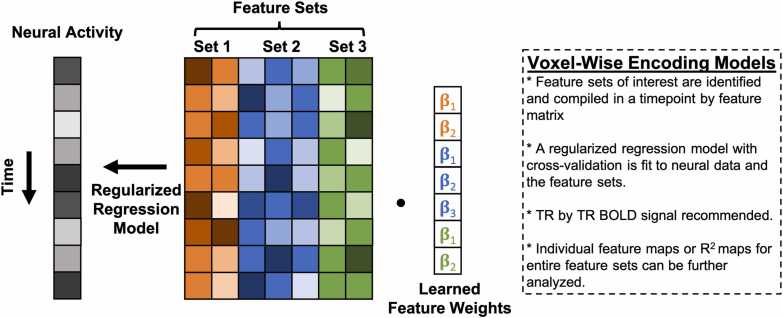
Voxel-Wise Encoding Model Schematic.

Implementation of MVPA often requires supporting auxiliary techniques and concepts that are not strictly part of MVPA but are nevertheless frequently needed to complete an analysis. Many of the auxiliary techniques and concepts relevant here (e.g., cross-validation) cut across various types of MVPA as well as other types of analyses in developmental cognitive neuroscience that the reader may already be familiar with. For efficiency, we summarize auxiliary techniques and concepts germane to MVPA in Table 1, and encourage readers to read through the table before the review of each MVPA variant below if they are not already familiar. Schematics for each MVPA type described here are depicted in Fig. 1, Fig. 2, Fig. 3, Fig. 4. We created these depictions to be rooted in the data structures that a user would work with when sitting down to run MVPA in the interest of clarifying practice. The various other review papers previously referenced also have useful figures and depictions, so we recommend the reader consult those figures in order to complement what we show here and flesh out their own understanding. In additional, several of the supporting citations in Table 1 have useful visualizations of auxiliary concepts (e.g., K-fold cross-validation).

**Table 1:** Key Auxiliary Concepts.

Concept	Definition	Most Applicable To…
*K*-Fold Cross-Validation	Cross-validation is an iterative technique originating from computer science that is used to train predictive statistical models. The purpose of cross-validation is to aid the analyst in selecting a particular type of statistical model (e.g., support vector machine vs logistic regression), tuning the hyperparameters of a particular model, or both, with the goal of optimizing predictive power. *K*-fold cross-validation is conducted by first splitting a dataset into *K* independent folds to be used as *testing* data. The procedure runs for *K* iterations. On each iteration, a statistical model is fit and/or tuned on all available data that does not belong to the *k*-th fold (*training* data). The model is then used to make predictions on the test data and its predictive accuracy is quantified and saved. This procedure repeats until each of the *K* folds has served as the test data. The aggregate predictive accuracy of each model type or model hyperparameter is used to guide the analyst in selecting the most predictive model. Sometimes, prior to cross-validation, the analyst may opt to withhold a small subset of data from the cross-validation procedure to serve as a final *hold out,* or *validation* set*.* The final model specification may be fit to this set to obtain the most realistic estimate of out-of-sample predictive validity. If an analyst wishes to select a model type *and* tune hyperparameters, they may opt to conduct *nested* cross-validation wherein an additional cross-validation procedure is nested within each iteration of the main cross-validation procedure. Traditionally, the training data on the *k*-th iteration would be split into *J* folds and an additional cross-validation procedure would run over the *J* folds within the *k*-th iteration of the supraordinate iteration to optimize the hyper parameter. The optimized hyperparameter would then be used to fit the model and quantify predictive accuracy in the *k-*th fold. This procedure would then be repeated for each of the *K* folds. *Additional Reading:*Varoquaux et al., (2017), *NeuroImage*	Decoding Analysis, Voxel-Wise Encoding Models
Searchlight Analysis	A searchlight analysis is a procedure for understanding the information contained in local patterns of brain activation. Searchlight analyses are conducted by moving a sphere with a user-defined radius over every voxel in the brain, extracting the local patterns of interest from within the sphere, conducting a pattern analysis of interest (typically decoding or representational similarity analysis), saving the statistic of interest (e.g., decoding accuracy, similarity coefficient) to the center voxel of the sphere in an output map, and then moving the sphere over one voxel to begin the next iteration. Searchlights are typically implemented on individual subject data, with the end result thus being a set of a subject-specific maps carrying some kind of pattern-related information (e.g., decoding accuracy, representational similarity). It is important to note that these maps are unlike traditional univariate subject-level contrast maps insofar that the datum for a single voxel contains information from the entire *local searchlight radius*. Analysts can perform group level statistics over these maps as they ordinarily would in the traditional univariate framework, such as taking a group average using a GLM or regressing the results onto individual differences. As with traditional univariate analyses, researchers must still correct for multiple comparisons while considering the intrinsic smoothness of the maps. *Additional Reading:*Etzel et al., (2013), *NeuroImage*	Decoding, Representational Similarity Analysis
Model Hyperparameters	Hyperparameters are components of statistical models that are used to control the behavior of the model output (i.e., prediction variance, magnitude of coefficients, scale of model) but do not directly index a relationship between the input and output variables. Examples of hyperparameters include λ in ridge and LASSO regression (downwardly penalizing regression coefficients) or γ in support vector machines (controlling the curvature of the decision boundaries). Because hyperparameters do not bear a relationship with the model’s data, they must be set manually by the analyst. Rather than arbitrarily or randomly choosing a hyperparameter value, it is typically recommended to use cross-validation to determine a hyperparameter value that results in an optimally predictive model. Default ‘rule of thumb’ conventions exist for certain hyperparameters (e.g., 1 / number of features for γ), though it is unclear how suitable these conventions are for cognitive neuroscience research. *Additional Reading:*Weaverdyck et al., (2020), *Social Cognitive and Affective Neuroscience*	Decoding, Voxel-Wise Encoding Models
Regularized Regression	Regularized regression is a variant of linear regression where the slope coefficients are variance stabilized. Variance stabilization is a procedure that helps constrain the sampling variability of a statistic. Variance stabilization in regularized regression occurs by downwardly penalizing regression coefficients towards zero as a function of the number of model parameters. This means that regularized regression exploits the bias-variance trade-off by introducing a relatively conservative, controlled degree of bias into the model to reduce sample-to-sample variability of model predictions and thus overfitting in service of producing a more generalizable model. Depending on the type of regularization (*l*_*1*_ or *l*_*2*_) some parameters may be penalized down to zero, serving as a de facto feature selection tool. The properties of regularized regression described above also make it suitable for handling highly parameterized models. *Additional Reading:*Lindquist et al., 2017, *NeuroImage;*Lindquist and Gelman, 2009, *Perspectives on Psychological Science*	Decoding, Voxel-Wise Encoding Models
Distance Metrics	Distance in the context of MVPA refers to the geometric proximity of data points to one another, typically in a multidimensional space. There are several distance metrics available to analysts, including Pearson’s *r*, Spearman’s *ρ*, Euclidean, Mahalanobis, City Block, and so on. Some distance metrics take vector magnitudes and element ordering into account (e.g., Euclidean) whereas others only focus on element ordering (e.g., *r*). Differences in results between such classes of distances can be substantively meaningful. Distance metrics based on correlations are computed by subtracting the esteimated correlation coefficient from 1. *Additional Reading:*Bobadilla-Suarez et al., (2020), *Computational Brain & Behavior*	Representational Similarity Analysis
Neural Signature	Neural signatures are maps of what distributed brain activity patterns should theoretically look like when one is engaging in a given mental operation. Concretely, they are whole-brain multivariate pattern maps wherein each voxel is assigned a weight that describes how strongly said voxel is recruited by a given psychological process of interest. Neural signatures are typically engineered using machine learning techniques (e.g., cross-validation) and are optimized to be sensitive and specific to the process of interest (e.g., high out-sample predictive accuracy for intended construct, not predictive of theoretically orthogonal constructs). While gradual adjustments have been made to the procedure described above since its introduction in two landmark studies (Wager et al., 2013, Chang et al., 2015), it is generically comprised of using cross-validation to build a predictive model of psychologically relevant behavior (e.g., subjective ratings of a construct, reward values) from a set of composite variables that are derived from a feature reduction of all whole-brain voxels. The specificity and sensitivity of the signature is then assessed with further methods such as correlating voxels of the potential signature with meta-analytic maps (or existing signatures) of other or comparable constructs, or using pattern expression scores to show convergent and discriminant validity with additional behavioral outcomes. Weights from the optimally engineered signature are back-transformed to voxel space and saved as a statistical map. The neural signature map itself renders an expectation of what brain activity resembles when the signature’s constituent cognitive process is evoked. This means the map can be used to derive a continuous, quantitative index of how well brain activity patterns observed under another context match the signature of interest and helps analysts infer how strongly the signature’s cognitive process is recruited in said other context. *Additional Reading*: Kragel et al., (2018), *Neuron*	Pattern Expression
Representation	A representation is a structured set of data that carries information about some property of interest and can be used in a downstream process. The key feature of a representation is that its structure adheres to a decodable pattern, meaning that aspects of the represented property can be inferred from it. In neuroscience, voxel or neural activity patterns encode information about stimulus features, even if this information is not explicitly accessed by the brain for behavior. More broadly, representations are not limited to biological systems—an image on a smartphone contains information about luminance, visual salience, and affect, while a social network encodes relationships that can inform models of social influence and popularity. While this definition is useful for empirical analyses, the precise nature of representations, particularly in relation to neural systems, remains a topic of ongoing debate (Baker et al., 2022; Roskies, 2021) *Additional Reading:* Krigeskorte & Kievit, 2013, *Trends in Cognitive Sciences*	Representational Similarity Analysis, Voxel-Wise Encoding Models

#### 2.1.1 Decoding

Decoding is perhaps the most canonical case of MVPA. Decoding analyses flip the traditional structure of fMRI analyses by modeling stimulus or task categories as a function of multi-voxel neural patterns (as opposed to the historical standard of modeling a voxel’s BOLD signal as a function of task or stimulus; Kragel et al., 2018). The basic idea is to determine the extent to which the voxels in a given brain region, or suite of regions, distinctly code for stimuli or task states of interest. Being able to successfully decode (i.e., predict or classify) stimulus categories or task conditions from multi-voxel brain activity implies that the brain is sensitive to boundaries among said categories or conditions. Crucially, because decoding analyses are typically conducted in a way that removes magnitude differences^1^ between conditions or stimulus categories they can reveal neural sensitivities that would otherwise go undetected with univariate approaches.

The oldest form of MVPA, decoding was first introduced in the landmark Haxby et al., (2001) study on face perception and the fusiform face area (FFA) using split-half correlations. In Haxby et al., (2001), the authors attempted to determine whether brain activity in the FFA distinguished between faces, houses, and other object categories (notably, while this study predicted categorical outcomes, other studies have decoded continuous outcomes). To determine whether they could decode the stimulus category from multi-voxel patterns, they divided their data into even and odd runs and then computed within- and between-category correlations of multi-voxel patterns across the run splits. The authors showed within-category correlations between run splits were markedly higher than between-category correlations, and that these correlations predicted category membership significantly better than chance.

Decoding workflows have since evolved beyond split-half correlations. Prediction of stimulus or task categories on the basis of multi-voxel patterns has become a central aspect of decoding. Out-of-sample prediction techniques originating from computer science and statistics are used to help establish the sensitivity of a brain region to task or stimulus boundaries (Mahmoudi et al., 2012; Varoquaux et al., 2017). Modern decoding workflows generally entail (i) preprocessing data in such a way that does not eliminate fine-grained spatial details (e.g., apply minimal or no smoothing, refrain from normalizing subject data to standard space if possible), (ii) selecting a statistical model that fits the desired research question (e.g., support vector machine, regularized regression, convolutional neural network; Liang et al., 2023), (iii) performing nested *k*-fold cross-validation to tune the hyperparameters and fit the model (Fig. 1), and then (iv) capturing the model’s overall accuracy by averaging model performance across folds. Decoding analyses are typically conducted within subjects at the run, block, or trial level (sometimes even at the level of each frame). Depending on the experimental design (e.g., event-related vs block design) and what one is predicting (e.g., decoding response time or choice behavior at the trial level compared to run-level means of neural responses to stimuli), some levels of analysis might be more preferable to others, but one could theoretically decode at several levels (e.g., decoding decision categories from trial-level multi-voxel patterns vs decision frequencies from run-averaged multi-voxel patterns). Analysts who wish to remove condition-specific or voxel-specific means should do so at step (iii) such that means are removed after the data are split into train and test folds, as to avoid test-train leakage during cross-validation. Mean removal varies widely across the MVPA literature, with some analysts removing means within condition and others removing means across voxels, or both (see Coutanche, 2013). Because mean removal subsequently influences how results are interpreted, distinguishing spatial variability from mean responses is a nuanced and important topic when conducting a decoding analysis (Davis et al., 2014, Hebart and Baker, 2018, Smith et al., 2011). A full treatment on technical details is beyond the scope of this review, but we strongly recommend that novel users review the relevant literature cited herein and carefully consider their approach to mean centering to best align their analyses and research goals.

There are several variants of decoding available to researchers. Researchers can deploy decoding as an ROI-based analysis, whether it be on a singular ROI (e.g., medial prefrontal cortex (mPFC)), a suite of discrete ROIs (e.g., mPFC and dorsal anterior cingulate cortex (dACC) separately), or a set of merged ROIs (e.g., jointly using mPFC and dACC voxels). If one is interested in searching for an optimally predictive subset of ROIs (Clithero et al., 2009) or estimating the unique predictive power of individual ROIs (Yin Wang et al., 2017), they may use a combinatorial approach that entails evaluating the predictive performance of various permuted subsets of ROIs (pooling across all voxels in the subset) or testing changes in predictive accuracy when adding an individual ROI to a set. Studies that are agnostic to the locale of an effect, wish to ‘let the data speak for themselves’ in a data-driven way, or have some reason to believe that pattern encoding may transcend traditional anatomical or functional boundaries, may pair decoding with searchlight analysis (Etzel et al., 2013). Finally, hypotheses about amodal neural representations (Awwad Shiekh Hasan et al., 2016; Peelen et al., 2010; Yin Wang et al., 2017) can leverage cross-classified decoding models to determine whether a classifier trained on neural responses from one modality (e.g., auditory) can decode neural responses from another modality (e.g., visual).

Group analyses entail averaging over subject-level decoding accuracies and then performing a test of statistical significance against a meaningful null value (e.g., chance-level). We note here that comparable decoding accuracies between any two participants are not necessarily indicative of comparable multi-voxel patterns between said subjects, though it is possible to perform decoding at the between-subject level (Weaverdyck et al., 2020). Because classification accuracies are bound between zero and one, some kind of variance-stabilizing transformation must be performed prior to traditional group-level analyses or one may opt to use non-parametric permutation tests. Researchers may also standardize or normalize classification accuracies for each subject depending on the research question (e.g., using ranks from combinatorial decoding, Clithero et al., 2009), though this requires careful consideration to ensure the standardized quantities still align with the research question one is testing.

##### 2.1.1.1 Interpretational limitations of decoding

While we touch upon limitations and caveats to all MVPA methods surveyed, we want to draw particular attention to some interpretational pitfalls that frequently accompany decoding in particular, especially since one of the motivating factors of this paper is about how MVPA can better uncover developmental processes by studying neural codes. With respect to decoding, analysts with the goal of probing neural codes must keep in mind that if certain circumstances in the underlying neural data are met, decoding results can create the illusion of a multi-dimensional neural code that does not actually exist (Davis et al., 2014, Hebart and Baker, 2018, Mur et al., 2009, Smith et al., 2011). This is because a number of factors ranging from within-person voxel-level variability to response amplitude can drive decoding results in the absence of a meaningful multi-dimensional code.

Some solutions exist to mitigate these limitations. First, one could try to remove any potentially confounding univariate response information (Coutanche, 2013). This is deceptively difficult, however, as ‘univariate response information’ could actually refer to different several features of brain activity, each of which rely on additional assumptions that can further complicate analysis and interpretation (see Hebart & Baker, 2018). A second option would be to follow the procedure proposed by Diedrichsen and colleagues (2013), wherein multi-voxel patterns are decomposed into independent components and sequentially added to a classifier that evaluates their incremental predictive accuracy. If adding more than one component enhances accuracy, then one can more confidently assert the presence of a multi-dimensional code. Third, one could simply opt to use RSA or encoding models (reviewed below) in lieu of decoding if the goal is to uncover a multi-dimensional neural code. As we note later on, because RSA assumes isotropic voxel-wise variance, one should theoretically find it difficult to observe significant results in a given brain region if stimulus-dependent geometry is not present and one is using an appropriate distance metric (e.g., correlation metric, Euclidean distance with de-meaning but without variance normalization; Botero and Kriegeskorte, 2024). Cross-validation and incremental goodness of fit tests used with encoding models also render relatively more informative tests of multi-dimensional coding.

#### 2.1.2 Representational similarity analysis

Representational similarity analysis (Kriegeskorte et al., 2008) is a technique designed to compare the structure or organization of information between two or more representations. RSA formally quantifies the degree of overlap in representations (i.e., shared information) by establishing a common geometry between representations based on the element-wise similarities comprising each representation. The most common and simplest form of RSA in cognitive neuroscience is a comparison between two modalities: neural activity and some kind of stimulus feature or behavioral rating (Fig. 2). However, RSA is quite flexible, and we later discuss more complex cases involving model-based representations, more than two representations, social networks, repeated measures, intra-modal (neural to neural) comparisons, deep neural networks, and more.

To introduce the driving concept behind RSA we consider the following illustrative example, conceptually adapted from Weaverdyck and colleagues (2020). Suppose one wanted to compare the urban layout between Madison, WI and São Paulo, Brazil. Doing so initially seems challenging because the two cities differ quite drastically in the scale of their land area. After all, at the time of publication São Paulo is among the ten largest cities in the world, and Madison is not even the largest city in its own state! However, differences in the scale of land area are not necessarily indicative of differences in urban layout. One way to compare the two cities in spite of these differences is to compare relationships among city elements. This could involve examining the physical distance between places such as city hall, the airport, the flagship university, the art museum, state capitol building, and so on, in São Paulo and then examining whether the relative pattern of pairwise distances between these places is preserved in Madison. In this example, the collection of places from each city symbolizes a representation of each city’s urban layoutand each place constitutes the individual elements of the representation, whereas differences in land area represent qualitative or scale differences between modalities that interfere with direct comparison.

It is not difficult to translate the example above to brains and behavior, replacing one city with multi-voxel patterns derived from conditions of an in-scanner task (e.g., viewing classmate faces) and the other with a relevant behavioral outcome (e.g., ratings of classmate popularity or likeability) or stimulus features (e.g., normative ratings of trustworthiness). This example illustrates the concept of *first-order* and *second-order isomorphisms* (Shepard and Chipman, 1970)*.* Because stimulus attributes across different modalities are difficult if not impossible to directly compare (i.e., first-order isomorphism), RSA instead compares the pattern of element similarities between the two modalities (e.g., neural activation and behavior). The degree of correspondence between the patterns of element-wise similarities (i.e., second-order isomorphism) for each modality is used to infer representational overlap, or similarity between representational structures. This is equivalent to comparing representational geometries between each modality (Kriegeskorte and Kievit, 2013) – determining whether the distance between objects in a low dimensional geometric space (e.g., 2 dimensions) is comparable between modalities.

In the context of cognitive neuroscience, RSA is also used to help reveal information coding. However, whereas decoding seeks to determine whether the brain is differentiating between categories of interest, RSA is frequently used to help uncover whether, or how, additional types of information are indexed in neural responses (e.g., social network distance encoded in neural responses to pictures of one’s classmates; Parkinson et al., 2017). RSA is conducted by first computing *representational dissimilarity matrices* (RDM), which quantify pairwise distances between elements within a given modality (Fig. 2). At the neural level, this entails calculating all pairwise distances between multi-voxel patterns of interest. Researchers most commonly use 1 - Pearson’s *r* or Euclidean distance as their distance metric of choice, though others are available (Bobadilla‑Suarez et al., 2020; Chatterjee, 2020). We recommend using a correlation based metric or de-meaned Euclidean distance (without variance normalization) following recent work showing that these distance metrics allow for better capture of stimulus geometry associations in underlying neuronal responses (Botero & Kriegeskorte, 2024). A second RDM consisting of dissimilarity among behavioral ratings or other stimulus features of the elements is calculated in a similar manner (e.g., differences in behavioral ratings of a relevant dimension such as animacy; differences in a stimulus property such as luminance or visual complexity). The unique off-diagonals of each RDM are vectorized and correlated .^2^ The ensuing correlation coefficient quantifies the degree of shared information between representational structures. RSA is usually conducted within individual ROIs, by using a whole-brain searchlight, or both (e.g., Chikazoe et al., 2014).

The procedure above describes RSA in its simplest form, but the method is highly flexible. For one, analysts are not limited to comparing two representational spaces. Multiple linear regression can be used to accommodate more than two RDMs (Freund, Bugg, et al., 2021; Parkinson et al., 2017), which allows analysts to quantify how strongly individual types of information are indexed into a focal representation. For another, although common examples of RSA often highlight the method as being useful for examining information related to categorical differences between stimuli (e.g., human vs insect, animate vs inanimate; Connolly et al., 2012, Connolly et al., 2016) or deconstructing the perceptual features of a complex stimulus (e.g., vocal recordings containing affective, acoustic, etc. features; Giordano et al., 2021), one need not be strictly interested in these applications to use RSA – different task features can be coded into an RDM and used in an RSA to parse cognitive task *states* when the brain is executing higher order cognitions (Freund, Etzel, et al., 2021). As an example, Freund and colleagues (2021) used RSA to model three theoretical components of a verbal color-word Stroop (1935) task where names of colors were displayed in various hues and participants were instructed to say the name of color and not read the word. The authors of this study constructed neural RDMs using individual trials for a suite of cognitive control regions and modeled them as a function of three hypothetical dimensions of the task: target status (whether two hue-word pairings shared the same hue), distractor status (whether two hue-word pairings shared the same word), and incongruency status (whether both words in each hue-word pairing were incongruent during the experiment).

Alternately, one can test whether external, real-world social information, such as geodesic distances from a social network graph, is encoded in the brain (Schwyck et al., 2023). RSA can be leveraged to investigate representational content in a top-down, theory driven way by testing the degree to which representations conform to theoretically-implied RDMs (Brietzke and Meyer, 2021; Courtney and Meyer, 2020), or RDMs implied by computational models (such as similarity between embedding vectors derived from deep neural networks). In such a case, an RDM is created based on theoretically implied, or model-implied, distances between individual elements. This approach lends itself to also testing non-theoretical hypotheses about how a representation is constructed by generating an RDM according to any hypothesis of interest. Going a step further, individual cells of an RDM can be extracted and piped forward into other analyses (e.g., using subject-level RDM cells as an individual difference variable). Notably, RSA does not always involve comparing representational structures from different modalities. Researchers have previously used RSA to evaluate representational structures acquired in the same modality, such as in the case of repeated measures (e.g., comparing two neural RMDs collected at different timepoints; Charest et al., 2014) or comparing subject-level neural representations to group-aggregate neural representations (e.g., quantifying representational similarity between one participant’s neural RDM and an average neural RDM from several other participants; Broom et al., 2024; Chavez and Wagner, 2020) – both of which might be particularly relevant for developmental cognitive neuroscientists. Finally, RSA can be applied to neuroimaging data that do not involve multi-voxel patterns (Finn et al., 2020; Guassi Moreira et al., 2021; Guntupalli et al., 2016; Katabi et al., 2023; Lee et al., 2017), to non-fMRI neuroimaging data (Costa et al., 2014; Giordano et al., 2021; Kaneshiro et al., 2015; L. Wang et al., 2020), and to non-imaging data altogether (Brooks and Freeman, 2018)*.*

As with decoding, there is flexibility in how individuals aggregate subjects for RSA group-level analyses. The most common approach is to perform RSA at the subject level in independently defined ROIs or with a whole brain searchlight, and submit the resulting individual values (ROI) or statistical maps (searchlight) to a group-level statistical significance test against a null hypothesis correlation of zero. The same procedure applies whether one is testing a correlation between two RDMs, or regression slopes from a statistical model with multiple RDMs. If testing a correlation coefficient, the analyst would need to convert the correlation value to a continuous value using Fisher’s *r*-to-*Z* (inverse hyperbolic tangent) transformation, or perform a non-parametric test. Sometimes RDMs may be constructed at the subject level using only between run information to avoid conflation with scanner drift, or task structure (M. B. Cai et al., 2019). Finally, if comparing individual RDM cells across participants, one must carefully consider whether scale differences between participant RDMs may affect results and choose to standardize (or not) accordingly. Additional practical details and applications of RSA to cognitive, social, and affective neuroscience can be accessed in other recent review papers (Freund, Etzel, et al., 2021; Popal et al., 2019; Weaverdyck et al., 2020).

We conclude here by noting a key distinction between decoding and RSA: their assumptions about the spread of voxel variance in multi-dimensional space. A single multi-voxel pattern (e.g., a vector of beta weights from a given ROI) represents a point in this space, while a distribution of such patterns defines the overall shape of voxel variance in multi-dimensional space. This shape is crucial for understanding differences between RSA and decoding. RSA typically treats variance as isotropic (i.e., equally spread in all directions) unless explicitly reweighted (e.g., via Mahalanobis distance), whereas many decoding approaches adjust voxel weights based on variance estimates. As a result, decoding can succeed by focusing on a small subset of highly informative voxels, even if the broader representational geometry does not reflect the stimulus structure. In contrast, RSA typically requires that stimulus-related geometry is preserved across the entire region to detect meaningful representational structure.

#### 2.1.3 Pattern expression analysis

Pattern expression analysis is a relatively novel variant of MVPA. Whereas decoding and RSA are deployed to directly understand local information coding among a set of brain activity maps with known category labels, pattern expression aims to leverage the high dimensional nature of multi-voxel patterns to perform reverse inference on unlabeled brain activity maps in a principled fashion (Wager et al., 2013, Chang et al., 2015). Univariate activity in a given brain region can only provide a coarse and impoverished reverse inference (Poldrack, 2006). If one observes an mPFC cluster, for instance, in an unlabeled map of brain activity, it would be quite difficult to surmise what cognitive process was likely being recruited because of the redundant and multi-flexible functional nature of the brain - after all, mPFC has been implicated in univariate studies of economic valuation, emotion regulation, and social cognition, among other psychological processes (Delgado et al., 2016; De La Vega et al., 2016). Multi-voxel patterns, by comparison, are high dimensional in nature and characterize brain function more granularly since each voxel effectively represents an additional axis along which brain function can vary (and therefore be differentiated). Measuring neural activity in this space is conducive to better identification of discriminable brain states to facilitate reverse inference. Thus, a pattern of brain activity identified in this space can be more informative in performing reverse inference. Pattern expression analyses follow this logic by examining the degree to which the patterns in a given brain activity map resemble a neural signature of a core psychological process of interest. More information on neural signatures is given in Table 1, but we cannot emphasize enough that neural signatures must be sensitive and specific to the psychological construct of interest to guard against inaccurate reverse inference (Jabakhanji et al., 2022).

Pattern expression analyses require at least two components: a neural signature map and a statistical map of brain activity. Neural signatures selectively tap a single core psychological process of interest (e.g., reward, guilt, subjective fear) (Chang et al., 2022; Speer et al., 2023; Yu et al., 2020; Zhou et al., 2021). Brain activity maps are statistical patterns of activation (typically whole-brain) estimated during some kind of psychological state of interest (e.g., viewing faces of familiar others, decisions from a risk-taking task, viewing pictures of appetitive foods), usually one that is theoretically undergirded by more than one potential psychological substrate. The analysis proceeds by taking the dot product between the voxels in the neural signature and those in the brain activity map (Fig. 3). The ensuing scalar value quantifies the relative similarity between the neural signature and brain activity map. In theory, greater values indicate that the measured brain activity is increasingly reliant on, or performing, the core psychological process indexed by the neural signature. Pattern expression is most useful when one’s research inherently begets the question of what core psychological processes undergird a psychological phenomenon of interest (e.g., “Are risky decisions intrinsically rewarding to teenagers?”, “How relevant is mentalizing when children behave prosocially?”).

The relative nature of pattern expression scores means that researchers frequently use them in research designs that provide some kind of comparison, broadly defined. This can occur in various ways. One can test for differences in pattern expression between two groups receiving different experimental manipulations, or between different populations (e.g., patients vs controls). Alternatively, one can evaluate differences in pattern expression across an experimental manipulation within-subjects. If one has research questions about multiple core psychological processes, they may compare within-subject paired differences of pattern expression scores obtained with two different signatures on the same set of activation maps (e.g., “is psychological process X or Y more strongly expressed?”). It is also theoretically possible to test whether a single signature is significantly recruited or not by way of comparing pattern expression scores to a ‘baseline’ or ‘null’ signature. This could be executed in one of two ways. One approach would be to adapt what is used with RSA in the cognitive neuroscience memory literature: taking some kind of baseline scan that is unrelated to the neural activity of interest and then using that to compare pattern expression values (Tambini & Davachi, 2019). A second option would be to construct null signatures by simulating biologically plausible null data (Markello and Misic, 2021), perhaps even applying the same neural signature to procedures used in spin tests wherein the cortical surface is inflated and randomly rotated to preserve local spatial autocorrelation. Pattern expression may be used in more complex designs, such as predicting trial-level behavior as a function of fluctuations in one (Doré et al., 2017) or multiple (Guassi Moreira et al., 2021) neural signatures. Such analyses may also be easily complemented with metrics of univariate brain activity (Cosme et al., 2020) to compare against the effect of response magnitude on behavior. Pattern expression scores may also be correlated with individual difference variables of interest (Baranger et al., 2025). Because pattern expression scores are effectively used as a summary variable in these contexts, group level statistical tests are carried out as they normally would be in each respective context (e.g., paired differences, correlation). Finally, although we have mostly discussed pattern expression as a means for understanding neural information coding via reverse inference, there is also a literature on using neural signatures and pattern expression as biomarker and predictive tools (Baranger et al., 2025; Wager et al., 2013). In this context, neural signatures and pattern expression are used in combination with predictive modeling to predict the likelihood of an individual reaching a diagnostic threshold (e.g., for depression or substance use), much in the same way as polygenic risk scores are used in genetics (Choi et al., 2020).

Practically, pattern expression analyses can be performed by transforming brain activity maps to the neural signature’s space (usually a standard space), or by transforming the neural signature to each subject’s native space. To our knowledge, there has been no formal evaluative comparison of both approaches, likely due to the prevailing MVPA convention to conduct subject-level analyses in native space and then transform any resultant maps to standard space for group-level analyses. Given the lack of knowledge in this area, paired with increased scrutiny on analytic flexibility in cognitive neuroscience and related fields (Botvinik‑Nezer et al., 2020; Nosek et al., 2022; Wratten et al., 2021), we recommend authors run and report analyses both ways for the time being. We argue this approach is appropriate for pattern expression analysis in particular because it is unclear whether the information of the activity maps or the neural signature should be given priority—applying a transformation to either will inevitably result in some loss of information but it is not immediately clear which is more consequential. Another point of consideration is centering, standardization, and normalization of activity maps (and neural signatures if using more than one signature). As with other analyses described here, there is no ‘one size fits all’ convention applicable to all studies. We encourage readers to think deeply about the approach that best suits their research question, and run and report sensitivity analyses as necessary.

A crucial point of consideration for pattern expression lies in the selection of the neural signature. The dominant approach to-date is to use a neural signature that has been engineered and validated following the procedure introduced in Wager et. al. 2013 and Chang et. al. 2015 (see Table 1), either by tapping an existing, pre-engineered signature or engineering one’s own signature. We refer to these types of signatures as predictive neural signatures. Alternative options consist of using a meta-analytic map, such as those automatically generated on NeuroSynth (Kent et al., 2024; Yarkoni et al., 2011) or NeuroQuery (Dockès et al., 2020), or using an unsmoothed univariate brain activity map (subject-specific, or group-level, e.g., Jabakhanji et al., 2022). The choice of a neural signature is critical – one’s inferences from pattern expression analyses will only be as strong as the neural signature. If a neural signature is not properly validated, then the ensuing inferences will be less precise and rigorous. Even a well-validated, bespoke signature is subject to weakness–for example, the task used to derive the neural signature may not fully or uniquely tap the psychological process of interest, or the task may not generalize across contexts.

##### 2.1.3.1 Points of overlap between decoding and pattern expression analysis

Depending on implementation, pattern expression analysis and decoding can share several commonalities. Conceptually, both pattern expression and decoding approaches seek to identify how neural information is encoded. Moreover, pattern expression and decoding can become statistically similar when one chooses to use a neural signature that has been engineered by training a model to predict stimulus properties or task conditions. In this case one is effectively applying weights from a pre-trained decoder to novel data. This means one could theoretically implement a pattern expression analysis that is purely an extension or recapitulation of a decoding model (Baranger et al., 2025, Jabakhanji et al., 2022). However, there are key differences between decoding models and how predictive neural signatures are engineered.

First, predictive neural signatures and decoding analyses vary in in their spatial scale; predictive neural signatures are very often constructed by applying some kind of spatial data reduction method to whole brain data. Decoding, by contrast, is most often conducted at the level of individual ROIs or using a searchlight, and does not leverage spatial data reduction methods. Model coefficients are therefore slightly different in their interpretation because they are either tied to spatially reduced, whole-brain data, or to voxels from a constrained spatial milieu. Second, neural signatures are, ideally, intended to be sensitive and specific to the constructs they are tapping, which is not a requirement of decoding models. For neural signatures, this is achieved by the emerging practice of neurometric validation (Chen et al., 2021; Chang et al., 2022), wherein a series of supporting validation analyses are conducted to verify whether the signature actually taps the construct of interest. For instance, one may check whether the neural signature yields high pattern expression scores on data obtained from a task tapping the same construct but different to what was used for engineering the signature; or, one may verify whether the neural signature itself evinces high spatial correlation with meta-analytic maps of related and unrelated constructs (e.g., Guassi Moreira et al., 2023). Decoding models, on the other hand, don’t share this criterion – in fact, certain decoding models (e.g., cross-classified) are estimated with the express goal of determining whether the encoding of information generalizes across domains. Third, and related to this second point, there is a notable conceptual difference between decoding and pattern expression. Decoding analyses are typically suited for understanding how discrete or atomic features of stimuli or cognitive processes are coded in the brain; pattern expression relies on the neural signature to understand how core psychological substrates are deployed in more complex or involved mental processes.

Finally, we note use cases of pattern expression analysis where one does *not* use a neural signature engineered with predictive modeling. The most common such case involves using meta-analytic maps (Kim et al., 2022), in which an analyst taps the results from a meta-analysis of studies that identify a psychological process of interest. Meta-analytic maps as neural signatures come with their own set of challenges when trying to establish their validity (e.g., finding an optimal voxel-level threshold, ensuring specificity, etc.), but early use cases appear to be promising (Guassi Moreira et al., 2023, Kim et al., 2022). In the case of using meta-analytic maps as neural signatures, pattern expression analysis and decoding are even more distinct. In fact, one could go a step further and use a group-averaged contrast map as a neural signature (Jabakhanji et al., 2022). Such a choice would need to be properly justified by data showing the suitability of the signature (early results indicate some conditions of suitability; Jabakhanji et al., 2022), but it underscores the point that pattern expression analysis is ultimately a distinct analytic method from decoding. It is entirely possible that future work may delineate a strong, formal mathematic link between the two methods, but for now we argue that a distinction between the two is helpful for practical and conceptual purposes.

#### 2.1.4 Voxel-wise encoding models

Voxel-wise encoding models are a type of MVPA more explicitly aimed at uncovering the population codes indexed in brain activity by attempting to directly model them. They take their inspiration from foundational neuroscience studies that systematically tested how different stimulus features affected neuronal firing rates (Barlow, 1982; Hubel and Wiesel, 1962; Wurtz, 2009). This work initially discovered that certain populations of neurons were tuned to specific stimuli and variations thereof (e.g., a subset of neurons responding to reward value of a stimulus with modulated firing rates depending on reward magnitude). In human neuroscience conducted with fMRI, voxel-wise encoding models are implemented by regressing trial-by-trial brain activity over a set of core stimulus features selected by the analyst (Fig. 4). The stimulus features may be extracted from the stimuli in a data-driven way or identified top-down via theory. Fitting these models can help reveal the patterns of activity across the entire brain that code for specific stimulus features by way of showing which features carry the strongest or weakest associations with brain activity, or by adjudicating between competing feature sets (does the brain collectively care about one set of features over another?). Voxel-wise encoding models are engineered to be predictive, so they also serve as generative computational models. These models are commonly used in conjunction with naturalistic fMRI studies such as viewing dynamic film clips (e.g., Huth et al., 2012, Huth et al., 2016) but can also be used on task-based fMRI (Tang et al., 2023).

Notably, voxel-wise encoding models and RSA share a common mathematical framework based on the second moment of the distribution of activation patterns (assuming Gaussian noise). A full review of this framework is beyond our scope (see Diedrichsen and Kriegeskorte, 2017), but the gist is that encoding models and RSA are each aimed at parsing the same representational space, but go about doing it differently. Voxel-wise encoding models estimate ‘first-order’ parameters (i.e., feature coefficients) that help characterize the distribution of multi-voxel activity in a geometric space defined by task conditions. The distribution (variance) of these coefficients in the aforementioned geometric space is theoretically ready for downstream read out and are thus of relevance for computational modeling. RSA instead directly compares multi-voxel activities to characterize representations in terms of each condition’s position in a space defined by the voxels. These two approaches can be marshaled to produce equivalent knowledge about representational codes (e.g., coefficient feature maps from encoding analyses can be used to compare representational similarity between task conditions) in the context of building computational models of brain activity. However, the first-order parameters are often of interest to researchers because examining the spatial extent of their patterns can uncover coding preferences across brain regions. This means that encoding models also share some similarities with decoding, but the former are generally more informative and sensitive than the latter (see Naselaris et al., 2011 for a review).

As noted, features for voxel-wise encoding models can be identified in a top-down or theoretically informed manner (e.g., affective stimuli rated along feature dimensions of various emotion categories), or can be generated in a data-driven way (e.g., extracting word or image embeddings corresponding to each stimulus from a pre-trained neural network). Feature sets are often large and correlated, necessitating regularized regression (typically ridge regression) implemented via K-fold cross-validation. Even if feature sets are relatively modest in size, regularization is desirable because it enhances the model’s generalizability, can handle highly correlated features, and is needed to produce equivalence with RSA if one so desires. As is customary with optimal model engineering using cross-validation, feature sets should be rescaled in some way–standardized (Z-scored) or normalized (re-scaled to fit between 0 and 1). Whether one is directly modeling the BOLD timeseries (e.g., from naturalistic movie-viewing; Meschke et al., 2023) or using average activity for a given trial (i.e., modeling a beta-series of trials, Rissman et al., 2004) will impact feature processing, as the former would require averaging features within the sampling window of each TR. For instance, if one were regressing word embeddings extracted from a podcast played in the scanner onto the corresponding BOLD timeseries, one would need to choose a method to align the word embeddings for all words heard within each TR window after accounting for hemodynamic lag, (e.g., by re-sampling the feature timeseries, or averaging the embeddings together within TR, Wang et al., 2023).

Once the feature set is defined and properly processed, one would fit the model using K-fold cross-validation. Depending on one’s goals, cross-validation may be used for optimizing for the best tuning parameter for the regularized regression, estimating the predictive accuracy of the model when predicting novel or held-out data, or helping evaluate different feature sets. Voxel-wise encoding models can be fit to TR-by-TR fluctuations in preprocessed BOLD data, or may be used on *t*-stat images representing average brain activity for each trial on a cognitive task of interest (e.g., beta-series). Voxel-wise encoding models can be fit to yield coefficient maps for each feature or a map of the variance explained (*R*^*2*^) by a feature set (the latter is usually the variance explained in held-out data, or predictive accuracy from cross-validation), contingent on one’s research goals. If the feature set is large and data-driven, such as with word or image embeddings, individual feature coefficient maps may be difficult or virtually impossible to interpret. Depending on the data-driven method of feature extraction, the coefficients may be reduced (e.g., via PCA) to enhance interpretability and subsequently visualized (e.g., using an inverse term frequency matrix as features, reducing the feature matrix into several semantic topics, visualizing composite coefficient maps for each topic).

Voxel-wise encoding models can theoretically be fit at either the individual or group level (the latter after concatenation across subjects). However, most applications in the literature fit them at the subject level and then either interpret subject-specific maps (e.g., Huth et al., 2016) or perform aggregation over subjects (e.g., Deniz et al., 2023, Im et al., 2025). As with other variants of MVPA described herein, it is recommended to fit the model to data in native space and then transform the resulting maps (feature coefficient or *R*^*2*^ maps) to standard space. Testing feature coefficient maps at the group-level can be conducted as one normally would otherwise. Researchers can plot the spatial distribution of coefficients in a color-coded way to show the dominant feature at each voxel in the brain. Contrast estimates resulting from these maps may also be tested, though these may be noisy depending on the statistical relationships shared among features between and within sets. Feature coefficient maps can be fed-forward to other analyses that seek to test individual differences in feature encoding (e.g., such as regressing age against feature coefficient maps, or discriminating between spatial patterns of feature coefficients). Testing maps of model fit requires comparison to a baseline model (e.g., testing features relevant for social cognition against basic perceptual features or nuisance features) or specifying a meaningful non-zero null value, since observed *R*^*2*^ statistics are virtually guaranteed to be greater than zero for uninteresting reasons. Notably, these maps should be estimating the predictive power of a fitted model on held-out data.

##### 2.1.4.1 Points of overlap between mass univariate analysis and encoding models

As with decoding and pattern expression analysis, there are important points of convergence between voxel-wise encoding models and traditional mass univariate analysis. We acknowledge one could argue that encoding models represent an extension of the standard univariate general linear model (GLM), given that both approaches rely on similar statistical frameworks. We mostly disagree with this characterization as it overlooks key distinctions.

First, at a conceptual level, mass univariate analyses typically do not test core stimulus features thought to comprise relevant encoding dimensions. They are instead used to estimate and compare mean activity for task conditions of interest. Feature spaces and accompanying coefficients in encoding models, on the other hand, often transcend experimental task conditions in ways that are usually not modeled in mass univariate analysis. These differences represent key points of departure in the foundational goals of each approach. Second, in practice, encoding models frequently employ regularized regression to evaluate the competing effects of various feature sets, which are often highly parameterized, underscoring differences in the statistical engines undergirding the two approaches. Further in this vein, by virtue of their reliance on cross-validation, encoding models are fit to be predictive and generative, or relatively more robust to overfitting at the very least. Third, analysts using encoding models often prioritize assessing the variance explained by a given feature set rather than testing contrasts between individual coefficients. This differs from mass univariate analyses, which generally fit a smaller number of predictors to the BOLD signal and focus on statistical comparisons of individual (or combinations of) slope coefficients. Collectively, this means mass univariate analyses differ significantly from encoding models in the fundamental articulation of their analytic goals as well as their scope. While it is reasonable to consider encoding models as sharing relatively more methodological overlap with mass univariate approaches than other MVPA techniques, encoding models nevertheless diverge from the mass univariate approach.

#### 2.1.5 Emerging MVPA techniques

Additional MVPA techniques have been actively developed in recent years. We list and briefly summarize three such techniques here to preview emerging advances. First, multiple groups have independently attempted to merge information about MVPA with functional connectivity, (Anzellotti et al., 2017; Yoo et al., 2019) or at least integrate a temporal component with MVPA (Hyon et al., 2020). These techniques apply the logic of MVPA to connectivity—namely correlating fine-grained patterns of spatial activity across brain regions over time. Because these approaches are consistent with the basic biological knowledge that neurons tend to organize in populations, they are promising for uncovering more precise and discriminating information. Second, our research group has introduced a method for quantifying the spatial variability of multivariate activity patterns using Gini coefficients (Guassi Moreira et al., 2019; Meredith et al., revision under review). We reason that differences in the topological shape of brain activity in a given cortical locale are meaningful markers of cortical organization and, in a developmental context, could suggest a kind of specialization—activity that is more tightly coalesced and less diffuse may be indicative of a stricter ‘division of labor’ among the underlying neurons (while acknowledging the standard caveats associated with the various biological sources underlying the BOLD signal). Finally, scholars have recently used neural networks to learn embedding spaces from subject-specific multi-voxel patterns (Busch et al., 2024; Lin and Thornton, 2024). This has the potential to facilitate more accurate between-subject comparisons and to better delineate relationships between the brain states or stimuli encoded by multi-voxel patterns.

## 3 What kinds of developmental cognitive neuroscience research questions is MVPA useful for?

Here, we provide an overview of what kinds of research questions in developmental cognitive neuroscience can be answered using MVPA. In doing so, we highlight existing MVPA studies as illustrative examples that can stimulate future analyses and investigations. Although the remainder of this section is split up by MVPA variant (akin to Section 2.2), we stress to readers that research questions highlighted for a given variant may also be interrogated with a different variant, depending on the specific goals of the analyst and configuration of the analysis.

### 3.1 Decoding

Decoding is especially helpful for research questions involving multi-flexible brain regions that have previously shown comparable magnitude responses between different conditions or psychological processes, but could still be encoding information in said conditions differently. This is useful for developmentalists who may have questions about how stimulus categories or mental states of interest become, or do not become, differentiated across age or developmental stage (e.g., puberty) in commonmulti-flexible brain regions such as the mPFC, amygdala, ventral striatum, lateral prefrontal cortex (lPFC), temporoparietal junction (TPJ), and so on. For instance, one could use decoding to probe how representations of similarly valenced, but distinct, emotion categories (e.g., fear, anger, sadness) become differentiated in mPFC or the amygdala across development (Kragel and LaBar, 2016) given that prior behavioral work has shown that the psychological granularity of these affective experiences increases dramatically with age (Nook et al., 2017, Nook et al., 2018). Decoding analysis could complement these behavioral results by showing whether neural differentiation precedes behavioral differentiation. Going a step further, just as one could do in a mass univariate framework, an analyst could pit chronological age against pubertal stage to test whether age or pubertal timing (or tempo) is a better predictor of emotion differentiation. Another example would be to test whether the presence of close social others (e.g., parents and friends) during youths’ judgment and decision-making (Do and Telzer, 2024; Do et al., 2020; Kwon et al., 2021; Telzer et al., 2015) can be detected from brain regions that have not historically shown such distinctions in a mass univariate framework. Such findings could point to the involvement of previously overlooked psychological processes in processing social influence on cognition in youths.

Finally, we also note that even though decoding may carry with it some complications in identifying multi-dimensional neural codes (section 2.2.1.1), it still has properties that render it less sensitive to between-subject variances (Davis et al., 2014). This is noteworthy because even if a ‘simple’ one-dimensional relationship between stimuli and neural responses exists, decoding is likely to be more apt to detect it than mass univariate analyses. This can potentially confer serious benefits with respect to the feasibility of single investigator led studies and studies on individual differences in brain-behavior relationships.

### 3.2 RSA

RSA can be used to determine whether different features of cognitive tasks change in their relevance over age and development. For instance, one can use stimulus feature RDMs to better isolate elements of cognitive control from a color-stroop task (Freund et al., 2021). This could be leveraged to answer questions about whether age-related changes in task performance are indicative of skill maturation or age-related differences in how ancillary task features are processed (Telzer et al., 2018). This can be extended to tasks that have multi-faceted cognitive components, such as both inhibition and working memory. RSA constitutes an elegant way to simultaneously model multiple cognitive components of a task and subsequently quantify the relative contribution of each component to brain activity in a given region, allowing for investigations in the developmental timing for different components. Additionally, because RSA allows for comparison of neural information across different species (Kriegeskorte et al., 2008), developmental cognitive neuroscientists can use it to test whether the encoding of information or cognitive states is conserved across development between human and non-human species or to test causal relationships between early environments (e.g., early life stress) and neurodevelopment. Last, RSA can allow developmental cognitive neuroscientists ways to test how broader environmental information, such as the density or configuration of one’s social network, might be used when parsing information related to different social agents or for predicting future social outcomes from baseline neural data (Zerubavel et al., 2015, Zerubavel et al., 2018).

### 3.3 Pattern Expression

Pattern expression can be used to make inferences about what kinds of psychological processes drive behavior across development. This can be used to test key theories in developmental cognitive neuroscience, such as system-based theories of brain development and behavior. Such theories posit that developmental differences in motivated behavior are driven by fluctuations in the potency of various psychological systems (e.g., risky behavior driven by value-based and cognitive control systems, Shulman et al., 2016, Steinberg et al., 2018). These theories often presume the neural underpinnings of these systems are modular, meaning that activity of the systems is coded as the magnitude response of discrete brain regions, yet we know from neuroscience that modularity to this degree is largely unrealistic (Langdon et al., 2023, Panzeri et al., 2015). Pattern expression can allow developmentalists a better, more sensitive way to infer what kinds of psychological processes are indexed into complicated cognitive operations, such as decision-making or self-regulation (e.g., Guassi Moreira et al., 2021; Cosme et al., 2020), by estimating the association between behavioral actions (e.g., a decision) and pattern expression scores derived from brain activity directly preceding behavior (e.g., cognitive control pattern expression right up until the decision is made). Pattern expression can also be used in conjunction with naturalistic designs (e.g., Richardson et al., 2018) to determine what kinds of mental states or psychological processes are being recruited when processing stimuli with high ecological validity such as dynamic film clips (Camacho et al., 2023). Finally, pattern expression scores may serve as relevant biomarkers for building predictive models of clinical symptoms (Wager et al., 2013) which has clear relevance for neuroscientific study of developmental psychopathology. Recent work shows that pattern expression estimates are more sensitive to individual differences and may be better employed in conjunction with BWAS approaches (Baranger et al., 2025).

### 3.4 Voxel-wise encoding models

Applications of voxel-wise encoding models in developmental cognitive neuroscience involve directly examining how population codes may change with age or developmental stage, such as quantifying the relative salience of visual vs social features in naturalistic stimuli (Im et al., 2023). That voxel-wise encoding models allow researchers to test between competing feature sets gives developmental cognitive neuroscientists avenues to more precisely test the concept of ‘developmental shifts’ within persons across time (e.g., Gee et al., 2013) in a comprehensive, model-based fashion. Relatedly, we believe voxel-wise encoding models spurring methodological innovation in developmental cognitive neuroscience by being able to tease apart psychological sensitivity to different task components with age (Telzer et al., 2018), not unlike we discussed Section 3.2 with respect to RSA. Rrecent work from the nexus of neuroscience and computer science raises the additional possibility of using feature coefficients from voxel-wise encoding models to decode external states (Gardner and Liu, 2019). While this is traditionally done to predict external stimuli from encoding weights (e.g., visual stimuli), we can envision adjustments to this approach that could result in another fruitful avenue for estimating ‘brain age’ (Dosenbach et al., 2010) in domain-specific (i.e., task-based) contexts.

## 4 Suggestions for integrating MVPA with the current analytic ecosystem in developmental cognitive neuroscience

The field of developmental cognitive neuroscience has grown to develop its own unique and rich analytic ecosystem. This includes leveraging large consortium datasets, brain parcellations, longitudinal modeling, naturalistic stimuli, and BWAS. We not only believe that MVPA, as implemented in other fields, can enrich developmental cognitive neuroscience, but we also believe it can be tweaked and integrated with other analytic practices in this field for added benefits. Below we outline four potential points of integration between MVPA and existing analytic practices in the field in hopes of spurring methodological innovation.

### 4.1 Updating large consortium dataset derivatives

Promoting greater adoption of MVPA in developmental cognitive neuroscience is likely best achieved by enhancing the ease of conducting MVPA in large, publicly available consortium datasets. These datasets, such as the Adolescent Brain and Cognitive Development (ABCD) study (Casey et al., 2018) or the Human Connectome Project (HCP; Van Essen et al., 2013), are comprehensive multi-site studies that recruit several thousand participants and are planned by a central consortium. Frequently, the publicly available fMRI components of these datasets are most often and most accessibly comprised of *derivatives,* outputs of common processing pipelines (Gorgolewski et al., 2016, Gorgolewski et al., 2017). In the context of ABCD, the most readily used derivatives are tabulated data consisting of comma separated (.csv) or tab separated (.tsv) files that contain various metrics of brain activity for each subject across a series of ROIs or parcellations (e.g., connectivity matrices, beta values from a univariate contrast analysis, white matter integrity, etc.). Voxel-level data are not included in tabulated data releases. As is apparent to the reader, this complicates the implementation of MVPA because a researcher would need to compute, download and store several thousand additional activity maps. If the preprocessing is not optimal for MVPA, then the researcher would need to download even more data. This is a considerable impediment to using MVPA in large consortium datasets given the sheer size and scale of such data.

We can think of several potential solutions to this problem. First, the most straightforward solution would be to simply provide researchers with subject-level tabulated data of basic MVPA analyses in the same way that univariate contrast estimates are provided. Practically this could consist of including tabulated decoding accuracies of task conditions per each parcel or ROI as part of data releases, just the same as how univariate activations are already provided. This approach easily lends itself to decoding and could be reasonably accommodated with RSA and pattern expression.^3^ Tabulated RSA and pattern expression results could be generated several ways. Tabulating RSA could involve uploading vectorized RDM cells at the parcel or ROI level (e.g., an individual subject spreadsheet would have rows corresponding to ROIs or parcels and columns corresponding to neural RDM cells of relevant task conditions or features). This would give analysts flexibility to compare neural RDMs against other RDMs based on task structure or theory, and allow for testing of neural representational structures across time. Alternatively, tabulated data could also include representational similarity values between individual trials and task features (as in Freund et al., 2021). Pattern expression results could be tabulated in a similar way, with pattern expression scores being computed on a parcel/ROI or whole-brain level with neural signatures pre-selected by the consortium following community feedback. It is true that MVPA typically necessitates more analytic decision points than univariate analyses, but this should not obviate its usage. In the context of ABCD, for instance, this issue could be handled by one of its advisory committees settling upon common analytic standards for the analyses in a way that is no different from how other features of brain activity are already tabulated.

A second solution is to update data repository infrastructure for storage and download so that the statistical activity maps needed for MVPA are more easily accessible. While this solution places relatively more of a burden on researchers by virtue of requiring ample storage space for thousands of contrast maps, it would nevertheless be a boon to those who wish to conduct MVPA. It is more feasible to download individual activity maps than to download raw or minimally pre-processed time series data (i.e., 4D Nifti files) and compute the maps on one’s own, which would likely require re-running a pre-processing pipeline congruent with best-practices in MVPA (i.e., no or minimal spatial smoothing).

A third and final option could be to tabulate voxel-level data within each parcel or ROI across all task conditions per subject such that each row in a summary spreadsheet corresponds to a given voxel and each column corresponds to a task condition of interest (meta-data about voxel coordinates would also need to be included). This would cause the number of files in an analyst’s storage space to balloon considerably, depending on the ROI set or parcellation, but would still be relatively accessible insofar that one would not need to store or work with many Nifti files.

### 4.2 Integration with BWAS

Brain wide association studies have become increasingly commonplace in developmental cognitive neuroscience over the past decade. BWAS studies are conducted by correlating one (univariate BWAS) or more (multivariate BWAS) features of neural activity or structure to a theoretically or clinically relevant behavioral phenotype. BWAS studies have applications for basic and translational science. With respect to basic science, BWAS can link brain regions to behavioral phenotypes to outline potential causal models for future testing or to adjudicate between competing theories. Translationally, BWAS can aid in building predictive models of psychiatric or clinical disorders that can be used for screening and help search for candidate mechanisms for interventions. Though initial optimism and expectations were tempered when it was discovered that BWAS – at least, univariate resting-state BWAS (Spisak et al., 2023) – requires sample sizes that far exceed historical standards in human neuroimaging (Marek, Tervo-Clemmens et al., 2022; Poldrack et al., 2017), neuroscientists, psychiatrists, bioinformaticians, and psychologists alike continue to pursue them in the context of large consortium studies.

Given that every type of MVPA discussed here can be configured to produce output at the subject level, it is not difficult to envision how they may be integrated into a BWAS framework. For instance, instead of correlating a univariate contrast between beta-weights with a behavioral outcome, one could just as easily correlate representational similarity among RDMs, pattern expression scores, or decoding accuracies from a given ROI or parcel with the outcome. Similarly, one could correlate voxels from feature coefficient maps (voxel-wise encoding) or searchlight maps with the behavioral outcome of interest. Moreover, multivariate BWAS could be accommodated by building a predictive model of a behavioral outcome from all the cells within an RDM, much in the same way that scholars use cells within connectivity matrices (Shen et al., 2017; Z. Wang et al., 2021; Yoo et al., 2018). A collection of decoding accuracies (or the cells of a confusion matrix from a decoding analysis) or several pattern expression scores could be similarly leveraged. Recent results are beginning to hint that such approaches offer greater statistical power for characterizing individual differences than existing BWAS approaches (Baranger et al., 2025).

### 4.3 Applying MVPA with brain parcellations

One way in which developmental cognitive neuroscience could enrich the literature on MVPA for other fields is by applying such analyses to parcellated data. Developmental cognitive neuroscientists have played a key role in delineating the areal properties of the human brain (Gordon et al., 2022; Hermosillo et al., 2024; Marek et al., 2019; Seitzman et al., 2019). This endeavor has shown that the brain’s intrinsic functional wiring lends itself to functionally homogeneous ‘parcels’ that blanket the cortical landscape. However, most applications of MVPA in cognitive, affective, or social neuroscience do not involve the use of parcellations (though there are exceptions, e.g., Schwyck et al., 2023) and instead use pre-selected ROIs or searchlights.

In our view, this is a missed opportunity for several reasons. First, the use of parcellations can result in better statistical power by way of conducting fewer comparisons. Second, because parcels are derived based on maximizing intra-parcel functional similarity, they act as *de facto* ‘spatial priors’ and thereby minimize (though do not eliminate) the risk that one misses encoding information that spans parcel boundaries. Third, parcellations can accommodate a fair amount of analytical flexibility (Bryce et al., 2021), both in the different types of parcellation schemes, variations of a given scheme, and the ability to transform parcels into native space, so an analyst can find a scheme best aligned with their research goals. Fourth, parcellation schemes are often implemented with grayordinates, which combine cortical surface area mesh vertices with subcortical voxels for even greater spatial precision (Jiang et al., 2015)^4^ and could thus enhance analytical accuracy. Finally, the taxonomic nature of parcellations acts as a *lingua franca* between studies and promotes cumulative science.

We can envision the integration of MVPA with brain parcellations in a number of ways. The most straightforward and widely applicable way would be for parcels to serve the same function as ROIs serve for decoding and RSA. Instead of conducting said analyses in ROIs, an analyst would simply conduct them in parcels of interest In fact, parcellations may actually be a more principled, or more informed, way of defining ROIs. The same could be said for pattern expression analyses (e.g., Baranger et al., 2025). or voxel-wise encoding models, though more thought would need to be devoted to those applications because those approaches usually emphasize the importance of widely distributed neural codes. Nevertheless, it would be possible to compute pattern expression scores in a handful of theoretically meaningful parcels or fit voxel-wise encoding models to select parcels of interest.

A second approach specifically related to pattern expression could be to engineer whole-brain neural signatures used for pattern expression in a parcel-by-parcel manner. That is, the neural signature engineering process is not performed on data from the whole-brain, but rather repeated for each parcel (akin to the GWAS in the polygenic risk score method; Choi et al., 2020). It is possible that this creates more statistically efficient neural signatures by introducing fewer correlated features into the model building process and results in ‘parcel signatures’ that can be used individually or as a collection. However, care would need to be taken when aggregating scores from a large collection of parcels due to the aforementioned unmodeled correlations between parcels.

### 4.4 Longitudinal modeling and MVPA

Developmental cognitive neuroscience is unsurprisingly known for its strong tradition of longitudinal modeling. Any of the MVPA methods described here can produce summary statistics that can be used as a variable in whatever longitudinal framework one wishes to employ (e.g., multilevel modeling, structural equation modeling, etc.). Whether it be decoding accuracies, representational similarity scores, pattern expressions, feature coefficients or feature set fits, MVPA can be straightforwardly integrated with longitudinal modeling. However, we want to use this section to highlight and propose more creative uses of MVPA in longitudinal data analysis, largely with respect to decoding and RSA (and a minor example with pattern expression).

In terms of decoding, one could leverage cross-classified decoding to determine how well a decoder trained at one time point can predict stimulus conditions or values at adjacent timepoints, ages, or developmental stages. This would be an interesting way of testing for different windows of neural differentiation across development. Relatedly, one could use combinatorial decoding over a pool of ROIs with repeated measures to predict a condition category or value at a final time point, helping localize which ROIs *at which time* contain the most predictive or discriminatory information.

RSA has similarly creative uses, such as comparing neural RDMs at various time points to a neural RDM from the final time point, and plotting the similarity to reveal any potential complex growth trajectories. One can also analyze RDM cells in a regression framework by predicting neural RDM cells concatenated across multiple time points as a function of interactions between complex or unique growth trajectories coded across several theoretical RDMs and time-specific dummy codes.

Finally, although we touched on the concept above, we reiterate that pattern analysis scores obtained from multiple, developmentally sensitive neural signatures (e.g., separate neural signatures of reward for ages 8–12, 13–18, 19–24, or a signature that dynamically incorporates interactive age effects) could render longitudinal data analysis of pattern expression more developmentally accurate.

We feel compelled to note that testing these new ideas should not come at the expense of forgetting recent lessons learned about other best practices involving reliability, effect size, and sample sizes in the context of fMRI analytics and longitudinal analysis (Brandmaier et al., 2018; Marek, Tervo-Clemmens, et al., 2022). It is important for researchers to carefully consider the anticipated effect sizes and reliability of the fMRI signal they are planning to analyze in order to inform sample size planning both in terms of scan time (*n*) and overall number of participants (*N*). Indeed, these issues should be considered for any imaging study, but are particularly relevant for longitudinal studies where a combination of modest effect sizes and poor reliability can hinder the detection of true patterns of change by obscuring the rank order between observations, making it impossible to distinguish meaningful change from error.

### 4.5 Enriching naturalistic fMRI experiments

Naturalistic fMRI experiments in the field can be enriched with the application of pattern expression and voxel-wise encoding models. Pattern expression analyses can be applied to bins of multi-voxel patterns along the course of movie watching to track how different psychological states are recruited over time. These data can be used to test whether youths of different ages or developmental stage use different psychological substrates to parse naturalistic stimuli. Further extrapolating, these pattern expressions could be combined with inter-subject correlation (ISC) by creating a timeseries of pattern expression values from several TR bins from the naturalistic stimulus and then conducting ISC analysis. This could reveal whether individuals who are more similar in age have more similar recruitment of neural signatures throughout the course of naturalistic viewing. Similar analyses could be conducted with voxel-wise encoding models, by fitting models at the subject level and then comparing feature coefficient maps to all subjects, showing which population codes are similar at which ages (e.g., feature set X has high inter-subject similarity from ages 8–12 and then feature set Y becomes more dominant across individuals in ages 13–18). Alternately, one can compare the explanatory power of different features during naturalistic viewing as a function of age or developmental stage (Im et al., 2023).

## 5 Practical considerations and software

Here we quickly highlight several practical considerations for developmental cognitive neuroscientists who wish to implement MVPA, hoping to raise awareness of key issues and encourage further reading on these topics.

First, we note that MVPA is best conducted with many runs. This is because the activity between voxels needs to be well distinguished and obtaining many high-quality runs enhances the signal-to-noise ratio for activity of each voxel. While there exists some research on requisite sample sizes for MVPA (Mumford et al., 2012; Varoquaux, 2018), more substantive research on this area of fMRI methodology is needed and as such we cannot offer definitive sample size guidelines. Anecdotally, we recommend against running fMRI studies consisting of scan sessions that collect only one or two runs of many different tasks. Instead, single-investigator-led studies should consult existing literature from adult studies with comparable tasks and/or plan to collect *at least* three or four runs of a given task with many trial types of each condition per run. The same concept applies for naturalistic studies (i.e., the more data, the better).

Second, and relating to the issue of multiple runs, existing research suggests that decoding and RSA may best be applied between runs, especially if trial order is fixed for all subjects. That is, in the case of decoding, one should conduct cross-validation at the run-level, or at least include samples from different runs in the held out or test data (Mumford et al., 2014). This can be applied to RSA in the form of computing RDM elements by correlating neural activity patterns between runs. In both cases this approach may be more appropriate because residual nuisance signals from within the same run are likely to inflate the rate of false positives (Mumford et al., 2014). If the optimal manner to conduct the analysis given study design is still ambiguous after consulting existing literature, we recommend running the analyses both ways and transparently reporting *all* results, as understanding analytic sensitivity is critical to evaluating the robustness of results (e.g., Botvinik-Nezer et al., 2020). See Dimsdale-Zucker & Ranganath (2018) for additional useful details on this topic.

Third, we recommend keeping analyses in native space as much as possible, consistent with traditional MVPA conventions, unless there is a good reason to the contrary. This is especially true for longitudinal analyses that may compare neural activity from growing brains. Extracting summary statistics (e.g., pattern expression values, representational similarity, decoding accuracy etc.) and using them in other analyses (e.g., a regression) obviates the need to put longitudinal neural data in the same space and thus risk losing information. An outstanding issue is whether co-registration of longitudinal data to a single high resolution anatomical scan (e.g., midpoint, baseline) or to wave-specific anatomical scans is consequential for MVPA. Absent a devoted, systematic methodological study on the matter, it would be helpful in the interim for individual studies to run analyses both ways and compare results. Our intuition is that using time point specific high resolution structural images will result in the least loss of information and should thus be encouraged.

Fourth, the evergreen issue of head motion is highly relevant to MVPA as head motion poses a threat to estimating clean, precise multi-voxel patterns. To our knowledge, there is no systematic methodological investigation that definitively settles the matter of how much motion is too much motion for MVPA, likely owing to the fact that MVPA is typically employed in adult populations that are better at keeping still in the scanner than children and teens. Absent this work, we recommend that developmentalists treat MVPA akin to resting state and use a strict frame displacement (FD) threshold of 0.2 mm or 0.3 mm (Siegel et al., 2014) for individual frames to censor and then encourage authors to control for mean FD values at a later analytic stage. Although developmental samples may be hard to acquire, we are adamant that authors should not relax this threshold beyond 0.9 mm. Admittedly, this recommendation is based on extrapolating from first principles for other methods, so we hope to see large datasets such as ABCD or HCP used in the future to more systematically probe this issue.

Fifth, if performing decoding analyses on task-based data, it appears that single trial activity estimates are preferred, relative to working with the BOLD signal obtained from pre-processed TR level data (Mumford et al., 2012).

Last, we note there are several viable software packages for running MVPA. The authors’ personal preference is to use python-based Nilearn (nilearn.github.io), which can easily extract multi-voxel patterns, implements various machine learning algorithms via the sci-kit learn module, has advanced plotting capabilities, and can run off Windows, OS, and Linux systems. Brainiak (brainiak.org) is another powerful python-based software that can run on OS or Windows via Docker. Brainiak requires additional libraries to read in data (e.g., nibabel for image manipulation^5^). Both libraries can perform other non-MVPA types of fMRI analyses. pyMVPA (pymvpa.org) and the Matlab-based CoSMoMVPA (cosmomvpa.org) softwares are alternatives to Nilearn and Brainiak. All four packages can implement searchlights, though we personally find the brainiak implementation to be simultaneously the most flexible and intuitive. Finally, the Himalaya python library (https://gallantlab.org/himalaya/index.html) is a tool specifically designed to efficiently fit voxel-wise encoding models to fMRI data via banded ridge regression. Most important of all, each of these software contains helpful tutorials and walk-throughs that are high in practical and technical pedagogical value. We encourage interested readers to sample multiple libraries and access their respective documentation.

## 6 Concluding remarks

In this review, we have surveyed four core MVPA approaches, clarified their utility for developmental cognitive neuroscience and outlined next steps and considerations for the field. The scientific questions of developmental cognitive neuroscience and its history of methodological rigor make it particularly well-suited for MVPA and we see this as a promising direction in the next chapter of the field.

## Author contributions

JFGM developed the review concept with critical feedback from JAS. JFGM drafted the manuscript with substantial feedback and critical edits from JAS. Both authors approved the final manuscript for submission.

## Declaration of Competing Interest

The authors declare that they have no known competing financial interests or personal relationships that could have appeared to influence the work reported in this paper.

## Data Availability

No data was used for the research described in the article.

## Footnotes

1 It is not strictly necessary to remove magnitude differences. Practically, however, analysts often wish to test whether stimuli categories are represented in neural activity beyond magnitude differences.

2 Some analysts prefer to use Spearman’s *ρ* for this step to avoid incomparable scales or to not assume a strictly linear relationship between representational structures.

3 The approach described in this paragraph would not lend itself well to voxel-wise encoding models given that voxel-level data are needed to conduct the analysis, and summarizing over parcels or ROIs would defeat the purpose of the analysis.

4 One notable drawback to using parcellations in grayordinate space is that they are aligned to a standard space, which could pose complications based on one’s goals and the exact type of MVPA one wishes to use.

5 Nilearn’s data reading capabilities are currently built on top of Nibabel, so one would not need to learn two syntaxes to toggle between both Nilearn and Brainiak
